# Elderly and aged asthma have different characteristics: results of a multicenter study

**DOI:** 10.55730/1300-0144.5792

**Published:** 2023-11-18

**Authors:** Ebru DAMADOĞLU, Özge ÖZTÜRK AKTAŞ, Bilun GEMİCİOĞLU, Nafiye YILMAZ, Fulşen BOZKUŞ, Vehbi AYHAN, Ayse Füsun KALPAKLIOĞLU, Ferda ÖNER ERKEKOL, Yavuz HAVLUCU, Fuat EREL, Ömür AYDIN, Aydanur EKİCİ, Ayşe BAÇÇIOĞLU, Serap ARGUN BARIŞ, Gözde KÖYCÜ BUHARİ, Berrin CEYHAN, Özlem GÖKSEL, Mehmet KÖSE, Adile Berna DURSUN, Füsun YILDIZ, Arzu YORGANCIOĞLU, Sacide Rana IŞIK, Dane EDİGER, İpek Kıvılcım OĞUZÜLGEN, Ahmet Uğur DEMİR, Gül KARAKAYA, Ali Fuat KALYONCU

**Affiliations:** 1 Division of Allergy and Clinical Immunology, Department of Chest Diseases, Faculty of Medicine, Hacettepe University, Ankara, Turkiye; 2 Division of Immunology and Allergic Diseases Ankara City Hospital, Faculty of Medicine, Ankara Yıldırım Beyazıt University, Ankara, Turkiye; 3 Department of Pulmonary Diseases, Cerrahpaşa Faculty of Medine, İstanbul University-Cerrahpaşa, İstanbul, Turkiye; 4 Department of Chest Diseases, Faculty of Medicine, Ataturk University, Erzurum, Turkiye; 5 Department of Chest Diseases, Faculty of Medicine, Sütçü İmam University, Kahramanmaraş, Turkiye; 6 Division of Allergy and Clinical Immunology, Department of Chest Diseases, Faculty of Medicine, Recep Tayyip Erdoğan University, Rize, Turkiye; 7 Division of Allergy and Immunology, Department of Chest Diseases, Faculty of Medicine, Kırıkkale University, Kırıkkale, Turkiye; 8 Department of Allergy and Immunology, Medicana International Ankara Hospital, Ankara, Turkiye; 9 Department of Pulmonology, Faculty of Medicine, Celal Bayar University, Manisa, Turkiye; 10 Department of Pulmonology, Faculty of Medicine, Balıkesir University, Balıkesir, Turkiye; 11 Division of Immunology and Allergy, Department of Chest Disease, Faculty of Medicine, Ankara University, Ankara, Turkiye; 12 Department of Chest Diseases, Faculty of Medicine, Kırıkkale University, Kırıkkale, Turkiye; 13 Department of Chest Diseases, Faculty of Medicine, Kocaeli University, Kocaeli, Turkiye; 14 Division of Immunology and Allergy, Department of Chest Diseases, Ankara Atatürk Sanatoryum Training and Research Hospital, University of Health Sciences, Ankara, Turkiye; 15 Department of Chest Diseases, Faculty of Medicine, Marmara University, İstanbul, Turkiye; 16 Division of Allergy and Clinical Immunology, Department of Chest Diseases, Faculty of Medicine, Ege University, İzmir, Turkiye; 17 Department of Chest Diseases, Faculty of Medicine, Lokman Hekim University, Ankara, Turkiye; 18 Department of Pulmonary Diseases, Faculty of Medicine, Cyprus International University, Northern Cyprus, Turkiye; 19 Koc Healthcare, American Hospital, İstanbul, Turkiye; 20 Division of Allergy and Clinical Immunology, Department of Chest Diseases, Faculty of Medicine, Bursa Uludağ University, Bursa, Turkiye; 21 Department of Chest Diseases, Faculty of Medicine, Gazi University, Ankara, Turkiye; 22 Department of Chest Diseases, Faculty of Medicine, Hacettepe University, Ankara, Turkiye

**Keywords:** Asthma, allergic asthma, allergic rhinitis, elderly, elderly asthma, rhinitis

## Abstract

**Background/aim:**

Characteristics of asthma in the elderly population is not well-known. The aim of the present study was to evaluate asthma in the elderly population, to compare disease characteristics between patients diagnosed <60 (aged asthma) and ≥60 (elderly asthma) years of age.

**Materials and methods:**

The study was a prospective, multicenter, cross-sectional type. A questionnaire was filled out to patients 60 years of age and over, that have been followed for asthma for at least 3 months. Asthma Control Test (ACT), eight-item Morisky Medication Adherence Scale (MMAS-8) was filled out, inhaler device technique was assessed.

**Results:**

A total of 399 patients were included from 17 tertiary care centers across the country. Mean age was 67.11 years and 331 (83%) were female. The age at asthma diagnosis was ≥60 in 146 (36.6%) patients. Patients diagnosed ≥60 years were older (p < 0.001), had higher education level (p < 0.001), more commonly had first-degree relative with asthma (p = 0.038), asthma related comorbidities (p = 0.009) and accompanying rhinitis/rhinosinusitis (p = 0.005), had better asthma control (p = 0.001), were using less controller medications (p = 0.014). Inhaler technique was correct in 37% of the patients with no difference in between the groups. Treatment compliance was better in elderly asthma patients (p < 0.001). In the multivariate logistic regression analysis, having well-controlled asthma (odds ratio = 1.61, CI = 1.04–2.51), and high medication adherence rate (odds ratio = 2.43, CI = 1.48–4.0) were associated with being in the elderly asthma group.

**Conclusion:**

The characteristics of asthma are different among patients aged 60 years and over which seems to be related to onset age of asthma. In our cohort, the elderly asthma patients had higher education level, and treatment adherence and asthma control was better. Patients diagnosed ≥60 years of age did not have more severe disease.

## 1. Introduction

The world population is getting older. According to World Health Organization (WHO); by 2030, 1 in 6 people in the World will be aged 60 years or over and the population over 60 years is expected to double by 2050^1^. The population over 65 is the fastest growing group of the US population [1]. Considering that the prevalence of asthma is similar in the elderly age group compared to the general population (5%), it is obvious that the number of asthma patients in the elderly population will increase [1].

Asthma is a heterogeneous disease, the characteristics and treatment response differ across age groups [2]. When compared to younger asthma patients, asthma morbidity and mortality were reported to be higher in the older patients, exacerbation risk is increased and older patients are hospitalized more commonly when admitted to the emergency department for an asthma exacerbation [2–4]. Asthma may be underdiagnosed in the elderly, comorbidities may complicate the course, and data on drug efficacy and adverse effects are limited as elderly patients are often excluded from studies^2^. Previous studies including elderly asthma patients mainly document characteristics of asthma in the elderly population or compare elderly asthmatics with the younger ones, or the age of asthma onset was before 60 years of age [1,5–7].

There is scarce data regarding the phenotypes of asthma, level of asthma control, treatment compliance and health care utilization of elderly asthma patients in our country. The aim of the present study was to evaluate the disease phenotypes in asthma patients aged 60 years and older, and to compare the clinical characteristics of patients diagnosed ≥60 years with those of the ones diagnosed before 60 years of age.

## 2. Materials and methods

The design of the study was a prospective, multicenter, cross-sectional type. Current and previous members of the Turkish Thoracic Society (TTS) Asthma Interest Group designed and conducted the study. Study announcement was made to other TTS members via e-mails. Centers that wanted to participate were included and the study protocol was shared and discussed with all the participating centers. The study was conducted between October 2015 and 2017. The consortium protocol was approved by Hacettepe University Ethics Committee (GO 15/583-15).

Patients who were ≥60 years old, have been followed up with the diagnosis of asthma for at least 3 months and currently using a regular medication for asthma, who gave written informed consent were included. Diagnosis of asthma was established based on GINA guidelines in which compatible clinical history with documentation of variable expiratory airflow limitation was required for the diagnosis^3^. Patients with the diagnosis of bronchiectasis, evidence of tuberculosis sequelae on chest imaging, interstitial lung disease, and a smoking history of ≥10 pack/year were excluded. Presence of a severe psychiatric disorder (other than anxiety or depression) or dementia were exclusion criteria.

Elderly asthma was defined as asthma that is diagnosed ≥60 years of age and aged asthma was defined as a diagnosis before 60 years of age. Comparisons were made between the characteristics of the elderly asthma (asthma diagnosis ≥60 years of age) and the aged asthma (asthma diagnosis <60 years of age) patients.

A data collection form was filled out by the treating physician in each center for each patient, which included demographic, clinical and laboratory characteristics of the patients. Asthma control test (ACT) was applied and the score was recorded. Atopy was defined as a positive skin prick test result or positive serum specific IgE (a value ≥0.35 kU/L) measurement to commonly tested aeroallergens. Inhaler device technique assessment was performed with a 5 step check-list by the treating chest physician. These 5 steps included answers to these questions: 1- Does the patient properly prepare the device for inhalation maneuver? 2- Does the patient exhale properly before the inhalation maneuver? 3- Does the patient inhale the drug as required in depth? 4- Does the patient hold the breath for the required time (at least 5 s) after the inhalation maneuver? 5- Provide if there are any other mistakes.

Compliance with the treatment was assessed with the eight-item Morisky Medication Adherence Scale (MMAS-8) which is a widely used, validated in Turkish, self-report questionnaire and has high validity and reliability [8–11]. Total score for low, medium and high adherence were categorized as < 6, 6.0–7.0, and 8.0, respectively. Copyright owner (Morisky Medication Adherence Research, LLC) gave written permission for the use of the MMAS-8 questionnaire in this research.

### 2.1. Statistical analysis

Categorical variables were expressed as a frequency. Continuous variables were checked for normal distribution with the Kolmogorov-Smirnov test and normally distributed variables were presented as mean ± standard deviation (SD), and as median (lower-upper quartile) values if not. Nominal variables were compared using the Chi-squared test, Fisher exact test and interval variables were analyzed with a t-test, as appropriate. A p value <0.05 was defined as statistical significance.

Association of elderly asthma defined as asthma diagnosed after 60 years of age and asthma control variables were analyzed in the Multivariate Logistic Regression Analysis models. Models were adjusted for sex, education status, smoking status (never and ever), family history of asthma and allergic diseases, and biomass exposure for 10 years or more. Asthma control level was assessed by well controlled asthma according to ACT score (score > 19), unplanned visit in the last year for more than once, severe asthma attack in the last year and medication adherence. Statistical analysis was performed using IBM SPSS v.25.0 for Windows.

Post-hoc power analysis for the recruited sample size of 399, with the assumptions of effect size as OR: 2.0, good asthma control among the elderly asthmatics as 0.6 and proportion of variation explained by other factors in the model (R2) as 0.2 yielded an estimated power of 0.85 and type error of 0.00001.

## 3. Results

A total of 17 tertiary care centers from different regions of Turkey participated, and 399 patients were included in the study. Mean (±SD, min-max) age of the patients were 67.11 (± 5.73, 60–91), and 331 (83%) were women. Of the patients, the age at asthma diagnosis was ≥60 in 146 (36.6%) patients. The mean (SD) duration of asthma was 4.97 (4.17) and 20.76 (11.74) years for the elderly and the aged asthma patients, respectively.

Compared to the aged asthma patients, the elderly asthma patients were older (p < 0.001), had a lower body mass index (p = 0.01), a higher education level (p < 0.001), more commonly had first-degree relatives with asthma (p = 0.038), had higher frequency of asthma related comorbidities (rhinitis, sinusitis, nasal polyposis, drug allergy, food allergy) (p = 0.009) and accompanying rhinitis/rhinosinusitis (p = 0.005), were using fewer controller medications (p = 0.014), were more commonly on low dose inhaled steroids (p = 0.005), and the rate of unplanned doctor visit was lower (p = 0.001). Asthma was well-controlled (ACT > 19) more commonly in the elderly asthma group (60.3%) compared to the aged asthma group (43.5%) (p = 0.001). More patients with aged asthma had ≥10 years of biomass exposure (p = 0.01), and montelukast use (p = 0.003). All 5 steps of the inhaler technique were correct in only 37% of the patients and were not different between the groups (p = 0.237). Each of the 5 steps in the inhaler technique assessment was similar between groups. The characteristics of the overall patients and compared groups are shown in Table 1.

Compared to the aged asthma patients, the elderly asthma patients more commonly reported that they did not forget to take asthma medications and they never or rarely had difficulty remembering to take all their medications (both p values were < 0.001). The medication adherence rate was higher in the elderly asthma group (38.4% vs. 17.0%). Results of the MMAS-8 are shown in Table 2.

Multivariate logistic regression analysis adjusted for sex, higher education, smoking, duration of biomass exposure, presence of a first degree relative with asthma, and asthma diagnosis age group revealed that well-controlled asthma, and high medication adherence rates were associated with elderly asthma (Figure).

## 4. Discussion

The results of our study show that among asthma patients over 60 years of age, the elderly asthma patients, although being slightly older and more commonly having asthma-related comorbidities, compared to aged asthma patients were using fewer asthma controller medications, were more adherent with asthma therapy, and their asthma was better controlled. Of note, the elderly asthma patients had higher education level, less biomass exposure, and they more commonly had a first degree relative with asthma which might all have contributed to the diagnosis of asthma after the age of 60 years.

Previous studies mainly compare phenotypic characteristics of older and younger patients with asthma [1,5–7,12,13]. Asthma in the older adults is heterogeneous and may have different phenotypes, it may be early onset, or diagnosed after the age of 60 years in which there is scarce data on the clinical characteristics. The Epidemiology and Natural History of Asthma: Outcomes and Treatment Regimens (TENOR) study compared the characteristics of patients 65 years or older with the patients 18 to 64 years of age [1]. In the TENOR study, compared to younger patients, elderly patients had less health care utilization, steroid bursts and emergency department visits despite worse pre and postbronchodilator FEV1. Older asthmatic patients had a significantly longer duration of asthma and were older at asthma onset, however, the TENOR study did not compare elderly asthma patients according to the age of onset of asthma in itself, and the mean age at asthma onset was 42.3 years in their elderly cohort, and all patients in this cohort had severe or difficult-to-treat asthma [1]. In our study, all patients were over 60 years of age and mean FEV1 was similar between the groups, however the frequency of unplanned doctor visits in the last year was lower among the elderly asthmatic patients compared to the others. Similar to our findings older patients in the TENOR cohort had better scores for the behavior/attitude domain of the Asthma Therapy Assessment Questionnaire, had better compliance, and reported fewer unscheduled office visits [1]. When collectively evaluated, possible explanations for having fewer unscheduled office visits may be that they behave more planned considering their higher education levels, and commonly having a first degree relative with asthma may have caused more concerns about their health and they may have acted more compliant.

A cohort study that used patient-reported data at baseline and at 1-year follow-up in the United States examined the reasons for a higher rate of hospitalization of older adults with asthma [14]. Chronologic age was not an independent risk factor for being hospitalized, more respiratory symptoms, worse general health and low education level were the other prevalent risk factors that predict hospitalization. In the multivariate analysis being less educated was a significant risk factor for hospitalization in this study, however researchers did not analyze the age of onset of or duration of asthma [14]. Contrary to this, in our study elderly asthma patients had higher education levels, although we did not evaluate the rate of hospitalizations for asthma, asthma control level was better among elderly asthmatics. A recently published study from high-income Nordic countries reported that a lower education level was a risk factor for uncontrolled asthma in subjects with adult-onset asthma which was a consistent finding with the observations made in children revealing that a lower level of parental education has been a risk factor for uncontrolled asthma [15,16]. In this study, even among daily inhaled corticosteroid users, the ACT score was lower in those with primary education compared to the ones with tertiary education level defined as more than 12 years of education [15]. We previously reported that having a higher household income, which is associated with education level, was the only socioeconomic factor associated with better asthma control among women [17]. Although all patients in our cohort were over 60 years old and belonged to the same generation, patients diagnosed with asthma at or above 60 years old had a higher level of education with better asthma control.

Diagnosis of asthma in the elderly is often difficult to establish for several reasons; older patients may under perceive or under-report their symptoms, may be falsely diagnosed as COPD, besides congestive heart failure may accompany and mimic asthma symptoms [17]. In our study, we think that having a higher level of education and a first degree relative with asthma may have contributed elderly patients to reporting their symptoms and seeking pulmonologist care. On the physician side, in addition to these factors, the presence of rhinitis/rhinosinusitis and low biomass exposure may have favored the diagnosis of asthma in elderly patients. Therefore, it is possible that elderly patients who have more severe diseases and past exposures (e.g., biomass exposure, occupational exposures) might more likely receive the diagnosis of COPD, and we in asthma clinics see milder cases of asthma in the elderly population. Considering all these explanations, we think that patients ≥60 years old may be underdiagnosed in the population. As stated by Global Initiative for Asthma (GINA) additional and alternative strategies are needed for the elderly^4^.

GINA did not make a recommendation for the age cut off value for elderly onset asthma, but roughly there may be two clinical presentations in the elderly; late onset asthma, and long standing asthma [7]. Although some studies suggest middle age or older, late onset asthma patients generally first have asthma symptoms at ≥ 65 years of age, however up to 40% will have first symptoms after the age of 40 years [6,7]. In most studies older patient population is defined as being over 65 years of age; however, World Health Organization defines older people as being over 60 years, especially in developing countries^5^. As Turkey is a developing country, we decided to take this cut-off point for the definition of older age.

There are some limitations of the study. We included patients with asthma who are over 60 years of age at 17 tertiary care pulmonology centers across the country. While our findings represent the patient profile admitted to these centers, the profiles at primary and secondary care centers may be different. The strongest aspect of the present study is that, while it was thought that elderly asthma would be more commonly uncontrolled, it was shown in the present study that elderly asthma patients diagnosed at an earlier age were the ones that more commonly had uncontrolled asthma. The higher education level of elderly asthma patients and the fact that their first-degree relatives had asthma may have resulted in their higher awareness of the disease. In this way, it may have been easier for them to be diagnosed and their treatment compliance may have been higher.

In conclusion, the characteristics of asthma are different among patients aged 60 years and over which seems to be related to the age of onset of asthma. In our cohort, elderly asthma patients had higher education level, and treatment adherence and asthma control were better. High medication adherence and better control of asthma may be related to the higher education level in the elderly patients. Patients diagnosed ≥60 years of age did not have more severe disease compared to the ones diagnosed at an earlier age in the present cohort.

## Figures and Tables

**Figure:**
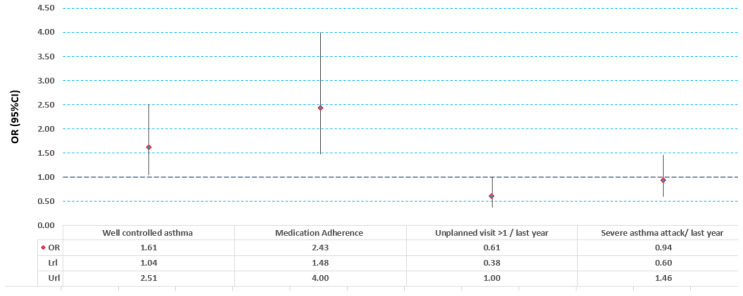
Association of asthma diagnosed age ≥60 years and asthma control variables in the Multivariate Logistic Regression Analysis.

**Table 1:** Patient characteristics.

n (%)	Diagnosis age ≥60 146 (100%)	Diagnosis age <60 253 (100%)	Total 399 (100%)	p
Mean age, years (SD)	68.92 (6.04)	66.06 (5.28)	67.11 (5.73)	**<0.001**
Duration of asthma, years, mean (SD)	4.97 (4.17)	20.76 (11.74)	14.98 (12.32)	**<0.001**
Asthma diagnosis age, mean (SD)	64.05 (4.70)	45.50 (10.47)	52.29 (12.55)	**<0.001**
Sex M/F	22/124	46/207	68/331	0.43
BMI, mean (SD)	30.64 (6.06)	32.45 (6.65)	31.79 (6.49)	**0.01**
High school/university graduate	43 (29.5)	19 (7.6)	62 (15.6)	**<0.001**
1st degree relatives with asthma	60 (41.1)	77 (30.6)	137 (34.4)	**0.038**
Asthma related comorbidities°	96 (65.8)	131 (51.8)	227 (56.9)	**0.009**
Hypertension	61 (41.8)	103 (40.7)	164 (41.1)	0.834
Accompanying rhinitis-rhinosinusitis	92 (63)	122 (48.2)	213 (53.4)	**0.005**
Aeroallergen sensitization#	40 (32.5)	66 (29.5)	106 (30.5)	0.626
FEV1%, mean (SD)°	79.82 (22.87)	78.43 (24.05)	78.95 (23.56)	0.58
Allergy prick test positive, (%)	38 (26)	62 (24.5)	100 (25)	0.19
Serum eosinophil count*, mean (±SD)	272.58 (233.59)	283.72 (300)	279 (273.79)	0.752
ACT >19	88 (60.3)	110 (43.5)	198 (49.6)	0.001
Use of >1 controller medication	135 (92.5)	247 (97.6)	382 (95.7)	0.014
Low dose inhaler steroid	48 (36.6)	39 (21.5)	87 (27.9)	0.005
Montelukast	54 (37)	133 (52.6)	187 (46.9)	0.003
Omalizumab	6 (4.1)	19 (7.5)	25 (6.3)	0.204
Systemic corticosteroid	1 (0.7)	9 (3.6)	10 (2.5)	0.08
Tiotropium	11 (7.5)	24 (9.5)	35 (8.8)	0.50
Beta blocker	23 (15.8)	23 (9.1)	46 (11.5)	0.051
Correct inhaler technique	60 (41.1)	88 (34.8)	148 (37.1)	0.237
Unplanned visit in last year, mean (±SD)	0.95 (1.28)	2.0 (3.70)	1.63 (3.12)	0.001
Unplanned doctor visit (>1)	34 (23.3)	94 (37.2)	128 (32.1)	0.004
Severe exacerbation last year	58 (39.7)	102 (40.3)	160 (40.1)	0.90
Severe exacerbation last year, mean (±SD)	0.60 (±0.94)	0.83 (±1.63)	0.75±1.42	0.11
Biomass exposure (≥10 years)	7 (4.8)	31 (12.3)	38 (9.5)	**0.01**

°comorbidities were rhinitis, rhinosinusitis, nasal polyposis, drug allergy, food allergy,

# skin prick test and/or specific IgE positive, performed in 347 patients,

* recorded in 249 patients

BMI: body mass index, FEV1: forced expiratory volume in the first s, ACT: asthma control test.

**Table 2:** Morisky medication adherence scale (MMAS-8)*.

n (%)	Diagnosis age ≥60 146 (100%)	Diagnosis age <60 253 (100%)	p

Do you sometimes forget to take your asthma medicine? No (1 point)	100 (68.5)	110 (43.5)	**<0.001**

People sometimes miss taking their medications for reasons other than forgetting. Thinking over the past 2 weeks, were there any days when you did not take your medication? No (1 point)	113 (77.4)	178 (70.4)	0.12

Have you ever cut back or stopped taking your medication without telling your doctor, because you felt worse when you took it? No (1 point)	112 (76.7)	198 (78.3)	0.72

When you travel or leave home, do you sometimes forget to bring along your medication? No (1 point)	108 (74.0)	168 (66.4)	0.11

Did you take your medication yesterday? Yes (1 point)	134 (91.8)	225 (88.9)	0.36

When you feel like your condition is under control, do you sometimes stop taking your medication? No (1 point)	101 (69.2)	164 (64.8)	0.37

Taking medication every day is a real inconvenience for some people. Do you ever feel hassled about sticking to your treatment plan? No (1 point)	111 (76.0)	170 (67.2)	0.06

How often do you have difficulty remembering to take all your medications? Never/rarely (1 point)	99 (67.8)	98 (38.7)	**<0.001**

- <6 (low adherence)	52 (35.6)	133 (52.6)	**<0.001**
- 6–7 (medium adherence)	38 (26.0)	77 (30.4)	
- 8 (high adherence)	56 (38.4)	43 (17.0)	

* The MMAS-8 Scale, content, name, and trademarks are protected by US copyright and trademark laws. Permission for use of the scale and its coding is required. A license agreement is available from MMAR, LLC., www.moriskyscale.com.

## Footnotes

1 World Health Organization (2022). Ageing and Health [online]. Website https://www.who.int/news-room/fact-sheets/detail/ageing [accessed 19 February 2023].

2 Global Initiative for asthma (2022). GINA Main Report [online]. Website https://ginasthma.org/gina-reports/ [accessed 20 February 2023].

3 Global Initiative for asthma (2014). Asthma COPD and Asthma – COPD Overlap Syndrome (ACOS) [online]. Website https://ginasthma.org/wp-content/uploads/2019/11/GINA_GOLD_ACOS_2014-wms.pdf [accessed 25 February 2023].

4 Global Initiative for asthma (2022). GINA Main Report [online]. Website https://ginasthma.org/gina-reports/ [accessed 20 February 2023].

5 World Health Organization (2002). Active ageing: a policy framework [online]. Website https://apps.who.int/iris/bitstream/handle/10665/67215/WHO_NMH_NPH_02.8.pdf?sequence=1&isAllowed=y [accessed 05 March 2023]
